# A Deep-Learning Based Visual Sensing Concept for a Robust Classification of Document Images under Real-World Hard Conditions

**DOI:** 10.3390/s21206763

**Published:** 2021-10-12

**Authors:** Kabeh Mohsenzadegan, Vahid Tavakkoli, Kyandoghere Kyamakya

**Affiliations:** Institute for Smart Systems Technologies, University Klagenfurt, 9020 Klagenfurt, Austria; vtavakko@edu.aau.at (V.T.); kyandoghere.kyamakya@aau.at (K.K.)

**Keywords:** documents classification, hard conditions, real-world conditions, deep convolutional neural network, document image processing

## Abstract

This paper’s core objective is to develop and validate a new neurocomputing model to classify document images in particularly demanding hard conditions such as image distortions, image size variance and scale, a huge number of classes, etc. Document classification is a special machine vision task in which document images are categorized according to their likelihood. Document classification is by itself an important topic for the digital office and it has several usages. Additionally, different methods for solving this problem have been presented in various studies; their respectively reached performance is however not yet good enough. This task is very tough and challenging. Thus, a novel, more accurate and precise model is needed. Although the related works do reach acceptable accuracy values for less hard conditions, they generally fully fail in the face of those above-mentioned hard, real-world conditions, including, amongst others, distortions such as noise, blur, low contrast, and shadows. In this paper, a novel deep CNN model is developed, validated and benchmarked with a selection of the most relevant recent document classification models. Additionally, the model’s sensitivity was significantly improved by injecting different artifacts during the training process. In the benchmarking, it does clearly outperform all others by at least 4%, thus reaching more than 96% accuracy.

## 1. Introduction

**Figure 1:**
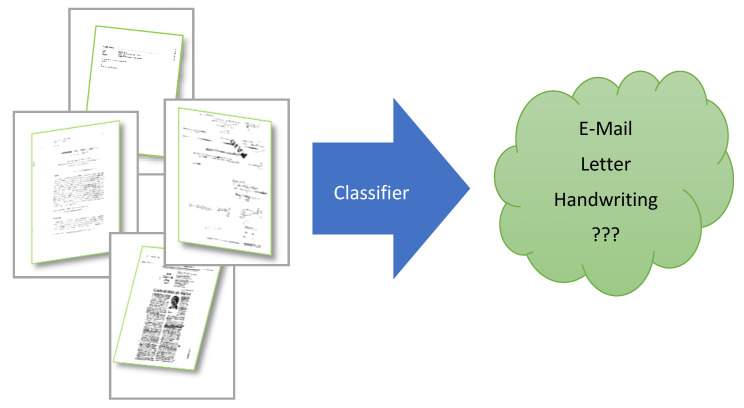
Document classification-related general processing pipe. The input of the classifier is a document image, and the classifier output is the estimated type/label of the input document.

Classifying images in general, including document images, is one of the most popular tasks in computer vision [1]. An image is classified based on special features included and not based on its structure. Following this definition, classification is indeed a sequential process that starts from preprocessing data, features extraction, features fusion, and finally assigns the input to one of the specified classes [2]. Figure 1 shows our core problem definition. Our model’s input is the image that contains one or more document images, and the output of our model is the label of the document(s) included in the input image. Some examples of document labels are Email, handwriting, a report document, bank card, ID, etc. Various factors or worse artifacts in the input images may significantly reduce the classification confidence. Some related examples: artifacts in the picture such as rotation, blur, shadow, or spotlight. 

Nowadays, visual sensors can perform more complex computer vision tasks as they now have more processing power. Therefore, one can see more use-cases related to object classification (document classification being just a subset). Some well-known real-world examples of such usages are image recognition and detection [3], emotion-sensing from face images, e.g., in the context of driver status monitoring [4], machine vision involving search and rescue missions using drones, performing video-based traffic control and surveillance [5], etc.

The management of hardcopy documents within an enterprise (a company) traditionally requires too much time and knowledge for the secretarial and archival staff to fully understand the various documents’ contents and classify them appropriately. This last-described task is not simple and generally consumes much time and is consequently proportionally expensive. Thus, an automated electronic classifier of (pre-scanned) document images (see “the digital office” concept) can reliably help to save both time and money for many organizations and companies [6]. Jointly with the increasing popularity of mobile phones, the almost pervasive use of integrated cameras for capturing document images is also increasing. The captured images depend on environmental conditions such as photograph experience, light conditions, etc. can have different qualities. However, the presence of various artifacts/distortions such as noise, shadows, and blur can significantly decrease the performance of a classifier [7] and eventually make it practically unusable (i.e., very low performing) in real-life conditions. Therefore, a robust (document) classification model for such hard/harsh scenarios is needed [8].

One can use various known image classification models to perform this document classification endeavor.

Indeed, whenever one has a classifier model containing different categories, it is not binary classification as the number of classes is more than one; the model should be optimized w.r.t to a related loss function for categorial classification. In this case, we peek at one of the most famous loss functions in this classification type, which was often used for classification tasks with different classes, the so-called categorical cross-entropy [9,10]. Equation (1) does present this chosen loss function:

(1) $$L\left(y,\hat{y}\right)=-\frac{1}{N}\overset{M}{\underset{j=1}{\sum }}\overset{N}{\underset{i=1}{\sum }}\left[y_{i,j}\log\left({\hat{y}}_{i,j}\right)\right]$$

where L is the chosen loss function with following parameters (Equation (1)); $y_{i,j}$ relates to the different expected labels, which is our ground truth; and The different observed labels ${\hat{y}}_{i,j}$ come from the model output; *N* is the number of class categories in our problem; *M* is the number of samples. The loss function will optimize weights in the model by reaching the minimum value during the training process. Subsequently, the model will be tested and verified with a defined loss function. 

For solving document classification, several traditional image classification schemes such as SVM (support vectors machine) exist to name one, theoretically [11]. However, various studies show that most of them are not robust enough to capture and learn the document classification task’s intricate patterns. Among those methods, some (e.g., SVM) were proven to be universal approximators for different problems. Therefore, one should involve many high-performing concepts to solve this challenging classification task at hand [11]. It is also proved that combining those universal approximator methods with dynamical neural networks such as cellular neural networks or convolutional neural networks can significantly improve performance. For example, Al Machot et al. [12] and Nasir et al. [13] show that combining SVM with cellular neural networks or convolutional neural networks considerably improves the SVM performance; therefore, a hybrid model can provide a robust detector/classifier instead of using the sole SVM model. 

Indeed, during the very recent years, several studies have tried to provide robust models for classifying document images or other types of images in the presence of various artifacts. Those studies can be grouped in two different approaches. In the first approach, the model consists of two separate modules: the first module does reverse the effect of artifacts and thus improve the quality of the image, and the second module is essentially a classifier [7,14]. The second approach is essentially based on augmentation. Thereby, the model consisting of one single module is trained by involving not just clean image samples but also with image samples that are artificially produced, on the fly, by adding (on clean image samples) random artifacts such as noise, contrast, etc. [15,16]. 

The deep neural concept developed in this paper involves CNN (convolutional neural network). CNN was first introduced by Yann LeCun et al. in the 1980s [17]. Despite several good advantages, it has not become popular on the first day. Indeed, in those days, most of the computers and single processing units and multi-core processors were not cheap. However, over the years, their respective costs have been significantly decreasing. Due to those lower prices, neural networks implemented on multi-core systems have been progressively increasing. Therefore, today, the usage of CNN can be found for solving various types of tasks related to data processing and classification such as sickness detection [18,19], image classification [20,21], street view image classification [22], remote sensing data classification [23], lidar image classification [24], data compression [25], and many other areas. 

The architecture of this network is based, as its name suggests, on convolution. Therefore, each convolution layer contains a kernel matrix, which is convoluted with that layer’s input, and the result will be passed through the activation function [21] to create the layer’s output. In the combination of those convolutional layers, some other essential intermediary layers are frequently used, such as sub-sampling (e.g., max-pooling) or “batch normalization” [21,26]. Those intermediary layers can be used after or before the convolutional layers. After several iterations of convolution and intermediary layers, the last layer will be connected to a “fully connected” layer. The number of layers required for solving problems is entirely open; however, increasing them makes the network deeper and provides more flexibility to solve a given problem. On the other hand, more deep layers will increase training time [27]. What makes the CNN model more powerful than traditional ANN (artificial neural network) lies in the first part of the model that tries to filter non-appropriate data. Therefore, decision-making will be easier and more accurate as the number of deep layers is appropriate. The input of the network can have multiple dimensions such as multiple images or a stack of different RGB channels with their blurred or edged images [28,29,30,31,32].

The model developed in this paper is constructed based on convolutional neural networks and does processes features extracted through a set of parallel preprocessing filters in the form of different input channels. In Section 2, we explain some relevant related works regarding document image classification. Our newly developed model is then described in Section 3. In Section 4, our model is tested\verified, and its performance is thereby compared with that of a selection of other well-known CNN models while involving the same test data for all. In Section 5, concluding remarks are formulated, which do comment virtually on the core results’ quintessence.

## 2. Related Works

There exist various image classification methods. However, our focus in this paper lies in the CNN-based image classification. It was introduced by LeCun et al. in 1998 [33] for classifying handwritten digits. This simple architecture model is based on three convolution layers, two average pooling layers, a fully connected layer (FC), and the output layer. The convolution layers use the sigmoid function for activation. The activation function adds nonlinearity to the system. The output layer has ten outputs corresponding to each of the possible classes from 0 to 9. The activation functions used in that model are Euclidian Radial Basis Function units (RBF) [34]. 

Two different models (LeNet-5, LeNet-5(with distortion)) were created based on the above-explained architecture. Both were trained and validated by using the MNIST dataset. The final models showed an acceptable accuracy performance of 95% and 80%, respectively.

Then, these previous models were studied and extended in various other studies. For example, it was proven by Geoffery et al. in 2006 that adding multiple hidden layers can improve classifier prediction [35]. 

Furthers, by increasing the power of processing units, a deeper neural network became feasible. Thus, a new deeper network such as the AlexNet was introduced. This model created by Krizhevky et al. in 2012 contained more layers compared to the LeNet-5 [36]. Additionally, a new activation function, the so-called “rectified linear unit (ReLU)”, was introduced. This new activation function makes training much faster than similar networks with other activation functions such as the sigmoid and tanh functions. Then, a new layer type performing a local response normalization (LRN) was introduced. The main role of this layer is to normalize data.

Also, among other related works for image classification, one can mention the new ways of classifying document images by analyzing the structure’s relationship, for example, through a statistical comparison of image patches in different segments of a document image. One interesting study was conducted by Kumar et al. [37] as they proved that combining the Relevance Feedback (RF) with Support Vector Machines (SVM) can improve classification accuracy. These combinations can better display their effectiveness when the number of features needed for classification is high. 

One of the main problems of CNN is that when problems become complex, the number of layers needs to be increased to cope with that complexity. However, that strategy has drawbacks. It decreases the training convergence. Hence, the time needed to converge towards the optimal weights is longer, and the required time for training is exponentially increasing with the number of layers. To solve this last-mentioned issue, He et al. [23] suggested making the models more straightforward by combining those layers into blocks and using those blocks, instead of layers, in the model. This idea was very satisfying, and it demonstrated that it could reach between 6 to 9 percent accuracy error on the CIFAR 10 dataset. 

This encapsulation’s success resulted in the creation of a more complex model by increasing and arranging those blocks’ functionalities. For example, in the so-called Squeeze-and-Excitation network (SENet), one can identify four functionalities within the blocks: (a) convolution, (b) squeeze, (c) excitation and (d) scale [38]. This new type of model displays far better results than the previous ones by reaching 2 to 3 percent accuracy error on top-5 classification.

Various methods of document image classification have been presented over recent years. Document image classification methods generally divide into two main categories, structure or layout based and content-based. This part provides an overview of meaningful work which have been presented based on the document classification. Kumar et al. [39] proposed a method based on statistics of patch codewords. It is initialized with a set of wanted and a random set of unwanted images, which the raw image patches extracted from the unlabeled images to learn a codebook. Spatial relationships between patches are modeled by recursively partitioning in the horizontal and vertical directions. Finally, a histogram of patch codewords is computed for each partition. In another work, Kumar et al. create a codebook of speeded-up robust features (SURF) descriptors based on some training images [40] and histograms of codewords [39].

Later, a Random Forest classifier was used for classification. That system performed reasonably even for limited training data; for example, we can mention Chen et al. [41], which propose a method based on Scale-invariant feature transform (SIFT) descriptors to classify documents. The approach only deals with structured documents, which are mostly text and with pre-printed contents. Joutel et al. use curvelet transforms as a multiscale method. This method is suitable for indexing linear singularities and curved handwritten shapes in document images [42]. Their way detects oriented and curved fragments at different scales and searches for similar handwritten samples in large manuscripts databases. Later, Kochi and Saitoh [43] compare the textual elements of document images. They showed that their system has good performance in shifts or noise in the target documents conditions and can handle semi-formatted forms. Bagdanov and Worring [44] classify machine-printed documents by using the Attributed Relational Graphs (ARGs). Byun and Lee use [45] partial matching for document structure recognition. This approach is limited to forms and is not generalizable to other document types. Shin and Doermann [46] compute geometrically invariant structural similarity. Their method scales to complete image matching (query by example) and sub-image matching (query by sketch).

Kevyn and Nickolov combine document layout and text features and verify their results by performing OCR on the retrieved documents [47]. Convolutional Neural Networks (CNN) were shown to cope with the structure-based document image classification tasks [40]. An important finding was that the learned deep representation is transferable across various jobs. Sermanet et al. [48] suggested using unsupervised pre-training, followed by supervised fine-tuning for pedestrian detection.

Similarly, supervised pre-training was proved helpful in different computer vision and multimedia settings w.r.t. a concept-bank paradigm [49]. Recently, Girshick et al. [50] showed that, for dealing with scarce data, supervised pre-training on more extensive data and then fine-tuning on smaller problem-specific datasets improves classification results.

In most of the related works, one can see that the models were trained and tested with datasets that do not contain artifacts such as noise, shadows, and blur. Therefore, those models’ performance does significantly decrease whenever they are used in real-life (hard) conditions [51]. Several studies have therefore underscored the need for robust models that shall work well both in real-life and in lab situations [52]. 

Two different strategies were suggested to achieve a strong model against various types of image degradation. In the first category, the classifier’s performance is improved by filtering the artifacts through an additional module [7,14,53]. In the second approach, the classifier model is made strong enough to classify degraded images without another pre-filtering module. Such a model is obtained through a more complex training strategy involving both a standard dataset of clean images and a further dataset consisting of images augmented by using/adding on clean images different artifacts that normally exist in the real-life usage domain [16,54].

For the first group of models, one can find many different models to classify images on specific artifacts such as noise or blur. For example, in the case of noise, a special kind of autoencoder (AE) called denoising encoder (DAE) is generally used to decrease the amount of noise in the image. Finally, the output of this pre-filtering module is fed into the CNN model for classification [7]. In the same way, for the blur-related degradations, the model tries first to guess the type of blur and then performs a deblurring of the image, and later on, the second part of the model is used to classify the deblurred image [55,56].

In summary, one can see that the document image classification endeavor is not an easy task. It needs complex neural models to better grasp the different aspects of the classification process, starting from features extraction to the selection of the correct class label. Various recent works show that CNN is a very flexible and reliable tool for solving this document image classification problem. However, the model’s accuracy and precision mainly depend entirely on the user’s model configuration. Hence, selecting suitable components of this model is a critical factor of success. On the other hand, most of the previous models are trained mostly only on lab condition pictures. Thus, they are not suitable for the difficult conditions considered in this paper. Indeed, the tough conditions (respective to the various distortions) call for a robust model, which can face such hard situations. For reaching this goal, we apply a two-step strategy. In the first step, we create by ourselves physical conditions to induce those various artifacts (at three quality levels) in camera-captured document images. A corresponding dataset is produced and used for the first training of our model. In the second step, we use Python libraries to artificially generate a bigger dataset mimicking those physically produced distortions; a much bigger dataset is generated for a better and further training of our model and thus making it more robust w.r.t. hard real-world distortions in document images. 

## 3. Our Novel Neural Model

**Figure 2:**
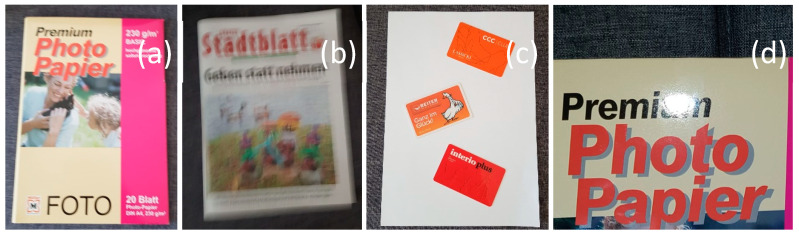
Main problems that can be encountered in document images: (**a**) Showing a document photo is usually taken from a mobile phone; (**b**) Showing a document image with motion blur; (**c**) Example of a document image delivering multiple documents within one single image shot; and (**d**) Example of a document image with a spotlight, which is blocking/disturbing reading the content. (Source: our own pictures).

As explained above, Figure 1 does show the overall goal of the CNN model to be developed in this work. There are some problems related to the quality of the input “document images” that need to be solved for reaching that goal: 

These problems can be grouped into four different categories (see Figure 2):

- Document image sizes, which are not always the same depending on the resolution that the scanning system (to generate a given document image) has had (scanner or smartphone camera) and on the document type (a bankcard generally has a much smaller size than a letter).
- Some images may have artifacts such as focus blur, motion blur, noise, spotlight, and shadows. We will have noise problems in shadows and low-light document images due to image sensor sensitivity problems.
- The documents’ orientation, which can be expressed in form of document rotations(s).
- The number of documents within one single document image can be more than one, especially for small documents such as identity cards, driving licenses, and bank cards.

**Figure 3:**
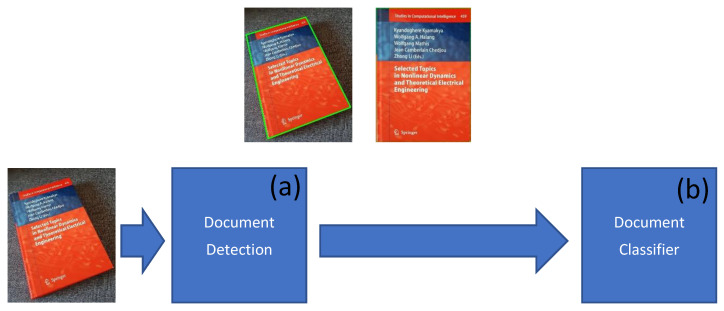
The novel global model is composed of two modules: (**a**) a Document Detection; and (**b**) a Document Classifier (Source: our own images).

For solving the mentioned problems, the following model (see Figure 3) was designed with two modules: (a) a document detection module and (b) a document classifier module.

The first module (document detection) is responsible for detecting the document’s boundary boxes inside the input document image. It has two different outputs: the first one shows a bounding box of the document image in the form of a quad polygon; the second is the probability of having a document inside the selected bounding box. These results (output of the first module) provide substantial help for the next module (classification). They enable good filtering of the input image by extracting only the useful part of the image where a document image is located and thus providing to the next module only the so extracted document image. The image will be cropped and transformed based on the list of detected bounding boxes (indeed, one may have more than one document image within the input image). The result list of document images is used to prepare the series of individual inputs of our second module (i.e., the classifier). 

### 3.1. Document Detection

In the scenarios of relevance for us, the input images involved in the classification do not contain just one document image; many contain more. For example, one can have, for specific reasons, a document image containing multiple cards such as a bank card, driving license, etc. For such a complex document image, one needs to detect and extract the different document images contained therein before being individually submitted to the classification module. 

**Figure 4:**
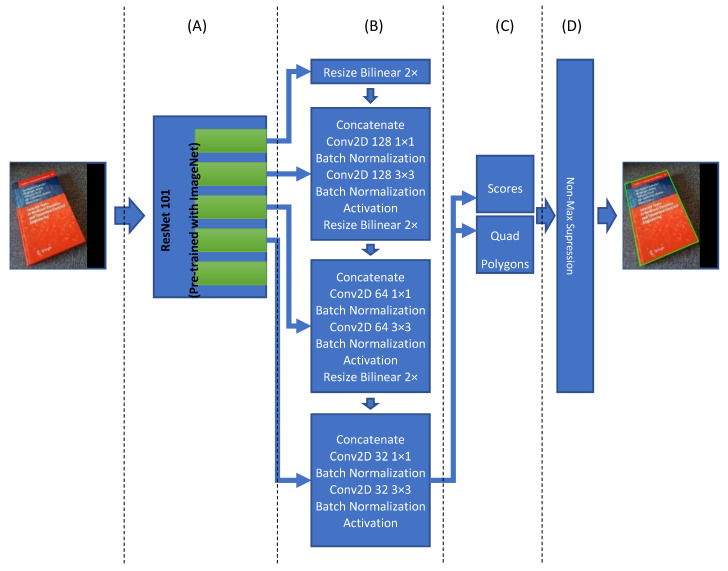
The document detection model contains three parts or sections: (**A**) Feature extraction based on ResNet101; (**B**) Further feature extraction layers; (**C**) Output layers; and (**D**) a Non-Max Suppression layer.

Figure 4 is showing the detailed architecture of this model. The input size of the model is fixed to 512 × 512 with RGB channels. Therefore, keeping the aspect ratio of the image is very important. For doing so, the maximum height or width of the image is resized to fit the 512 pixels while keeping the aspect ratio. This resizing process contains empty areas either on the right side or in the lower region of the input image; those open areas are filled with empty values (i.e., zeros). This model’s output is a list of quad polygons and their respective probabilities to find optimum bounding boxes. The final list of boundary boxes will be used to create our document image list for respective classification. Each of them shall be cropped from the original input image and then transformed as an individual input image for the classifier module.

The “input image” for the different classifier models described in the next part and the other related models involved in the benchmarking process are shown in Section 4. 

The document detection model is based on the “An Efficient and Accurate Scene Text Detector (EAST)” model [57], and it is appropriately customized to be used for our purpose. It contains four main parts: feature extraction, feature fusion layers, output layers, and finally, a Non-Maximum suppression layer. The feature extraction layers involve a ResNet101 network. The original EAST model uses a pre-trained PVANet network, but other studies show that it does not provide the required accuracy and precision [58]. Therefore, it is replaced with a ResNet101 network. The ResNet101 will extract features, and they will be used in the next part. Feature fusion layers, the outputs of the last four ResNet101 blocks, are used as input of the feature fusion layer. The first block’s output is resized two times in bilinear mode, and then it is concatenated with the fourth block of ResNet101. The output of the concatenation is the goes through some convolution, batch normalization, and activation layers. This process is repeated for other blocks’ output of the model until the second block of Resnet101 (see Figure 4B for more details). Finally, the last block of the feature fusion is used to create scores and related quad polygons. It is this convolutional neural network architecture that is finding the document boundaries. The last part of this model architecture is responsible for creating the final quad polygon boundary boxes and make the final boundary box based on the Non-Maximum Suppression (NMS) Algorithm with a 0.80 overlap threshold. 

The designed model is trained by augmented document image samples, which are artificially modified: rotated, warped, adding noise, adding blur, adding contrast. The dataset augmentations do enable reaching a much better accuracy in selecting documents from a given input image. After that training, the trained model was tested with 1000 real images from document photos, and it has shown 93.8% accuracy. After extracting the output, which is generally in the form of a “quad polygon,” this original quad polygon is perspective-transformed from a warped quad polygon into a square size of 256 × 256. 

### 3.2. Document Classification 

The second module of our global architecture (see Figure 3) is responsible for document classification. The main problem faced by this module is related to the document sizes, which are not always the same depending on the resolution that the scanning system or the smartphone camera (to generate a given document image) has had, on one side, and on the document type (a bankcard generally has a much smaller size than a letter), on the other side. In the first module, as already explained above, document images are rotated to correct the document’s possibly non-orthogonal orientation inside a given input image. Then, the extracted (in the first module) image portion containing a document image will be scaled to the size of 256 × 256 pixels. This enables the model to be suitable for any type of document image classification as most of the document images in various datasets have a rectangular form. The input image is provided with three (color) channels. The output of this module is a class number. 

To develop the best model for document classification, we created five CNN models to select the best suitable one for this module. These different models are described in this section.

#### 3.2.1. Model I

**Figure 5:**
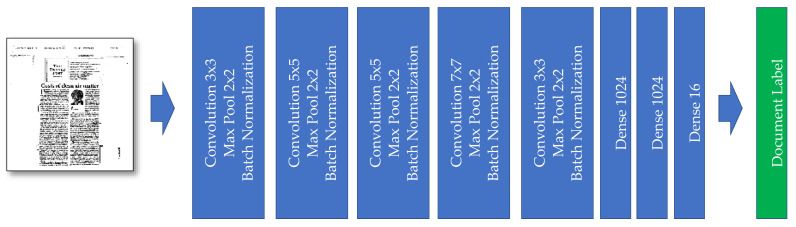
The “Model I” developed for document image classification.

Our first model is entirely a classical CNN model. The input of the model, as already explained, is an image with the size of 256 × 256 pixels. Combining convolution, max-pooling, and batch normalization layers are repeated five times to create the model. In the last section of the model, we have fully connected layers connected to those convolutional block outputs. The last dense layer has 16 outputs, the same as our number of classes (see Figure 5).

#### 3.2.2. Model II

Our second model has the same features extraction as the previous model. Thus, it has five convolution blocks, and each block is composed of convolution, max-pool, and batch-normalization layers. The last part also contains a fully connected neural network with three different layers. The final dense layer is responsible for creating outputs (i.e., labels). The number of neurons in this layer is the same as the number of classes (16).

**Figure 6:**
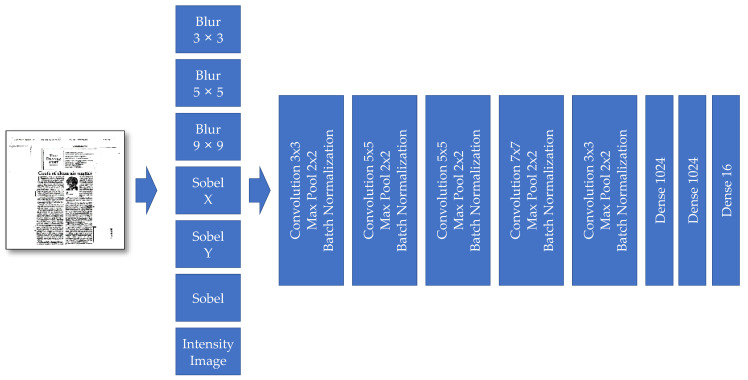
The “Model II” developed for document image classification.

These second model’s preprocessing layers provide more details and are indeed new channels besides the input image’s primary three-color channels. These new additional channels are, respectively: Blur 3 × 3, Blur 5 × 5, Blur 9 × 9, Sobel Filter X, Sobel Filter Y, and Intensity (see Figure 6).

**Figure 7:**
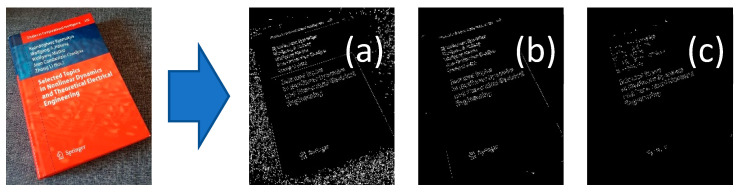
Effect of different blur filters on the image. For a better demonstration, a canny filter is applied on each blurred image to show image differences: (**a**) Blur filter with a kernel size of 3 × 3; (**b**) Blur filter with a kernel size of 5 × 5; and (**c**) blur filter with a kernel size of 7 × 7.

Those new preprocessing layers are extracting different information from the image. Figure 7 shows a canny filter effect to find edges within the image after a blur filter. The additional features can be captured by using different blur filters. The book’s background texture can be seen in image (a) of Figure 7, but it is not visible in other images of Figure 7.

#### 3.2.3. Model III

**Figure 8:**
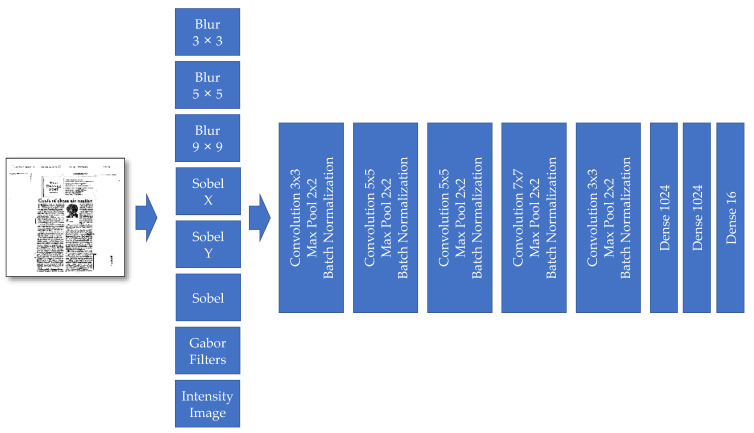
The “Model III” developed for document image classification.

Model III also has two main parts: (a) preprocessing layers and (b) features extraction layers. The feature extraction preprocessing layers contain different well-known filters, such as the following ones: Blur, Sobel, and Gabor filters (see Figure 8). Compared to Model II, the preprocessing layers from Modell III and the “Gabor filter” are added. These new preprocessing filters help the model in focusing on aspects of the input image, critical and relevant for the classification task.

**Figure 9:**
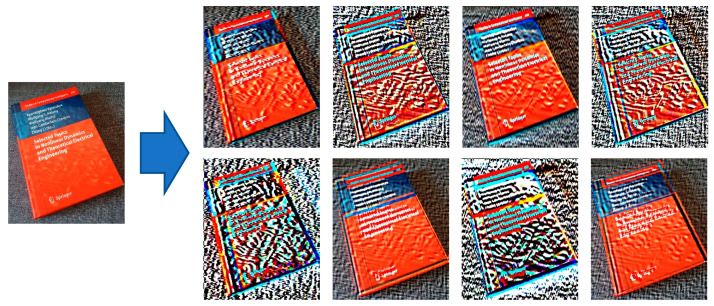
Example of Gabor filter outputs while playing with parameters, as one can see different output patterns are created. From left to right, the theta(rotation) is changed from 0 to 135 degrees. From top to down, the sigma is changed from 16 to 40. The kernel size which is used in this experiment is 40. (Source of input image: own images).

The role of preprocessing filters is to highlight the exciting parts of the model. Therefore, the central feature extraction has a better choice to select the required features from those parts. In this model, we additionally added the Gabor filter (see Figure 9). It is visually evident that each of those filters with changing parameter settings is spatially selecting different parts of an image.

#### 3.2.4. Model IV

**Figure 10:**
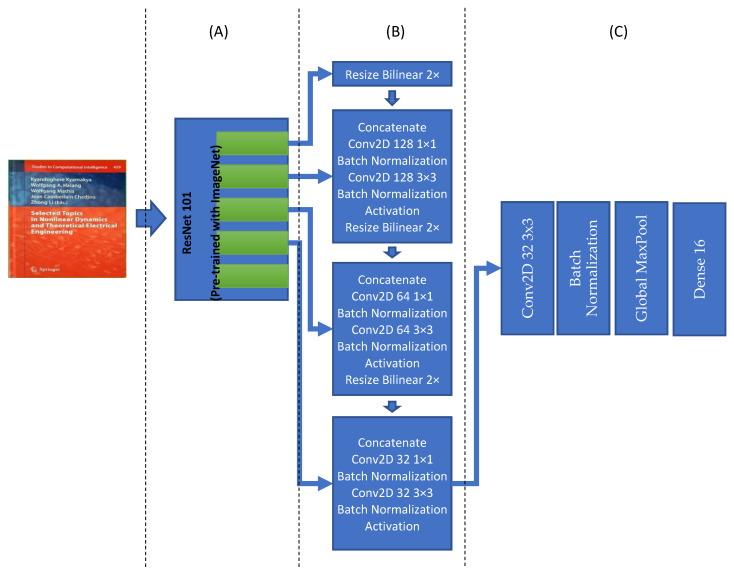
Model IV, it is containing three sections: (**A**) Feature extraction based on ResNet101; (**B**) Feature extraction layers; (**C**) Output layer.

Model IV, for document classification, is entirely different from the previous three models as this model will grasp various features but with the same goals as the last three models. This document classification model is based on the “Efficient and Accurate Scene Text Detector (EAST)” model [57], and it is customized to be used for our document classification purpose. It contains three main parts: feature extraction, feature fusion layers, output layers. The feature extraction layers have the ResNet101 network. The original EAST model uses a pre-trained PVANet network, but previous studies show that it does not provide the required accuracy and precision. Therefore, it is replaced with the ResNet101 network [58]. The ResNet101 will extract features, which will be used in the next part of the architecture. Feature fusion layers, the outputs of the last four ResNet101 blocks, are used as input of the feature fusion layer (See Figure 10).

#### 3.2.5. Model V

**Figure 11:**
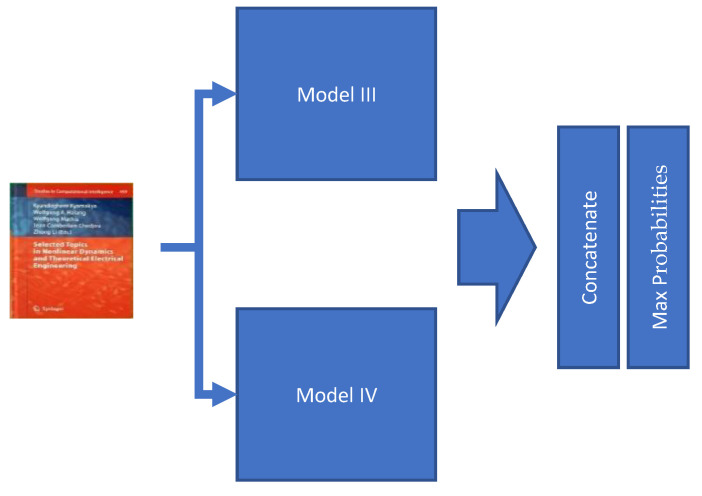
Model V does integrate two of the previous models, namely Model III and Model IV. First, it does classify the document image separately by the two integrated models and then finally concatenates and calculates maximum probabilities to determine the most probable class of the document image.

The last model, Model V, is the combination of two previous models, Model III and Model IV. Those two models are used in parallel to classify the document. The two obtained classification results are then combined based on higher scores (Figure 11).

## 4. Results Obtained and Discussion

For testing our model, a dataset was taken from A.W. Haley et al. [59]. A total of 160,000 samples were selected for training, whereby 40,000 samples were used for validation, and 40,000 samples were used to test the model. Both test and training have two different sections. In the first part, the model is trained with sample data with any artifacts, and the novel model is compared with previous studies with test images without artifacts. 

The second part is tested with noise, blur and contrast changes, and combined effect. One can see the model’s performance in the real simulated condition when one takes a picture from the mobile phone. The sample data consist of 16 balanced classes. Those 16 classes are the following: *letter, form, Email, handwritten, advertisement, scientific report, scientific publication, specification, file folder, news article, budget, invoice, presentation*, *questionnaire, resume, and memo*. Please note that some other similar databases exist, such as the dataset “Tobacco 3480”, but they have a significantly lower number of classes, namely ten classes instead of 16. The number of images is relatively much smaller.

The test machine used for paper is a PC with the following settings: Windows 10 Pro with latest patches, Intel Core i7 9700K as CPU, double Nvidia GeForce GTX 1080 TI with 8 GB RAM as GPU, and 64 GB RAM. Here, the training takes approximately 5 h for model III and 21 h for model IV. For model V, training is not needed; thus, training is not necessary here.

### 4.1. A Comprehensive Benchmarking of Our Different Basic Own Models 

To understand and find an objective justification of why our models best outperform the other ones, we conducted a simple feature significance analysis. For this purpose, we do use the so-called NMI (normalized mutual information) for the input features. Table 1 does show the Normalized Mutual Information (NMI) scores obtained for the input features, as involved in the different basic Models: Model I, Model II, and Model III; Model V is derived from the previous two ones (Model IV, Model III). Table 1 demonstrates that the NMI is significantly increased by adding more specific features using the multi-channel preprocessing units. 

**Figure 12:**
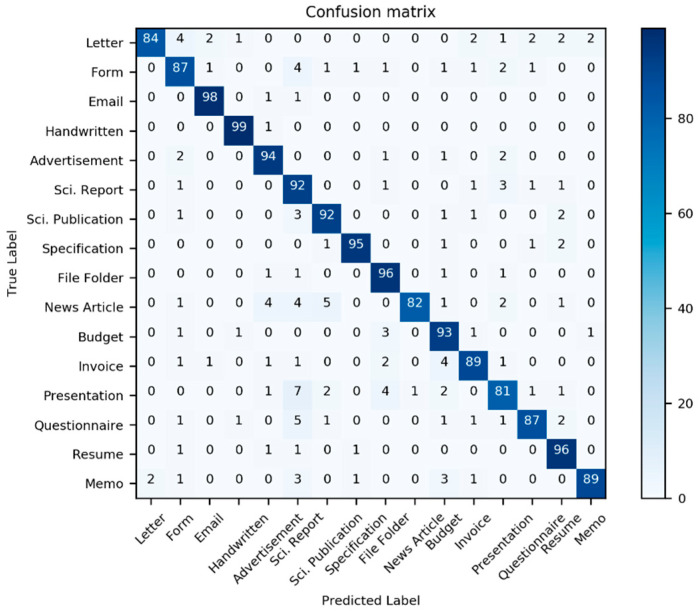
Confusion matrix of the performance results of model III for 1600 test data from the dataset Haley et al. [59]. (Source: our own images).

**Figure 13:**
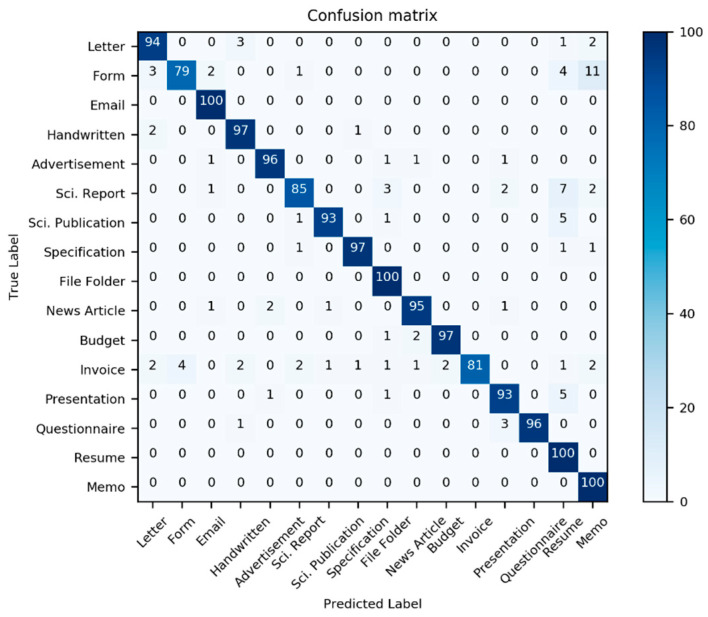
Confusion matrix of the performance results of model IV for 1600 test data from the dataset Haley et al. [59]. (Source: our own images).

**Figure 14:**
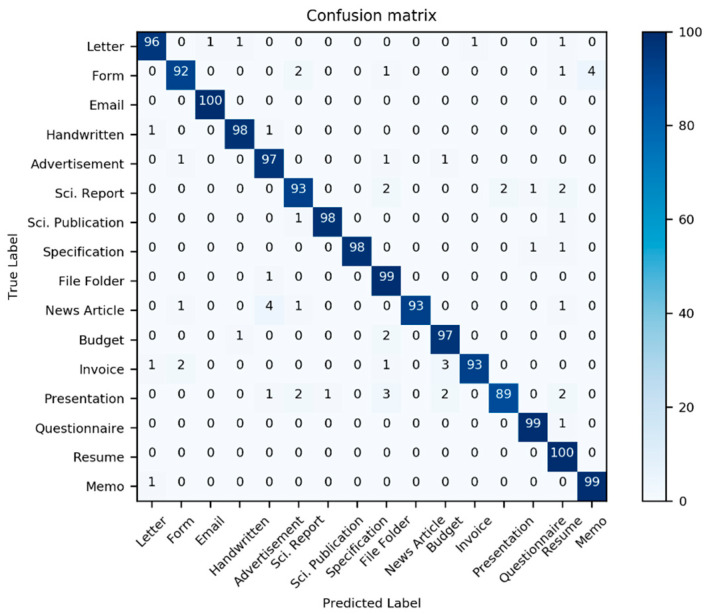
Confusion matrix of the performance results of model IV for 1600 test data from the dataset Haley et al. [59]. (Source: our own images).

Table 2 presents the classification performance scores for the three models in Table 1; additionally, two new models derived from the three previous models are also considered. Here, we use the well-known multi-dimensional classification performance metrics, namely accuracy, precision, F1-Score, and recall, to compare models comprehensively. Most classifiers have an interference problem with the class “Invoice”; it is often mistaken with other document classes (see Figure 12, Figure 13 and Figure 14). 

In Table 3, our novel classifier model’s performances are compared to some very relevant previous works. The accuracy and the false-negative ratio (FNR) are us as performance metrics. The results do clearly shown that our novel Model V (which does involve the above discussed multi-channel preprocessing features extraction) has the best performance compared to the various other models from the relevant recent literature.

Overall, two core strategies were used to significantly increase the final model’s classification performance, i.e., Model V. First, a set of preprocessing layers for feature amplification was used, followed by feature fusion layers. The second strategy has consisted of combining two different classification models in parallel, which has resulted in a much further increase in classification accuracy. This strategy’s success was confirmed by the experimental results obtained, see Table 3. 

### 4.2. Stress-Testing Our Novel Model (Model V) under Tough Simulated (Reality Emulating) Conditions 

**Figure 15:**
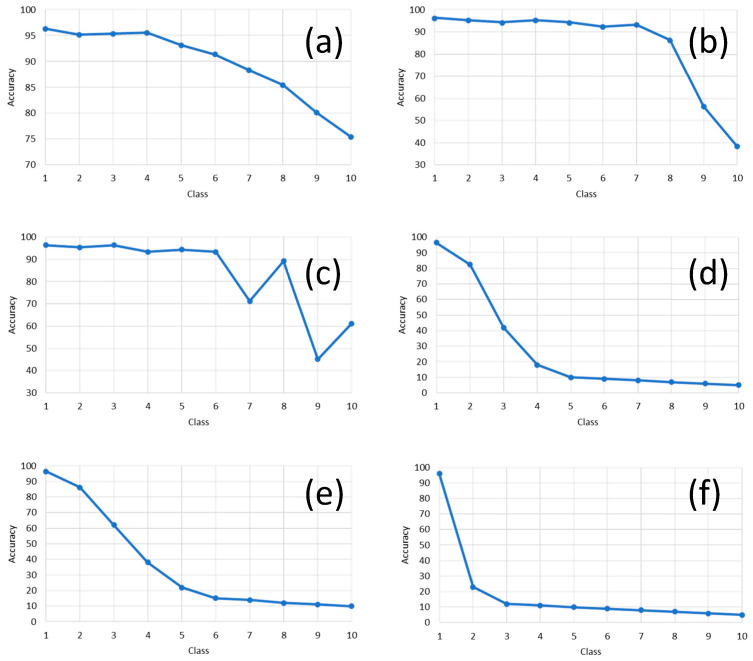
The effect of different artifacts on our best classifier’s accuracy (Model V). The class value shows the amount of injected distorting artifacts in the original clean image based on the parameter sets presented in Table 4. Scenarios: (**a**) Effect of Gaussian noise injection; (**b**) Effect of contrast change injection; (**c**) effect of brightness change injection; (**d**) effect of focus blur injection; (**e**) effect of motion blur injection; and (**f**) Effect of combined artifacts injection.

**Figure 16:**
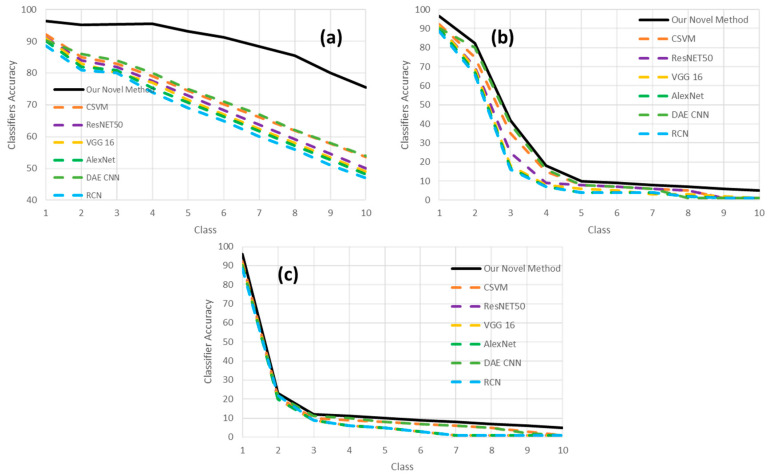
The effect of different artifacts on our best classifier’s accuracy (Model V) and a comparison with other related works. The class value (on the x-axis) shows the amount of injected distorting artifacts (i.e., distortion level) in the original clean image based on the parameter sets presented in Table 4. Three scenarios are considered: (**a**) Effect of Gaussian noise injection; (**b**) effect of focus blur injection; and (**c**) Effect of combined artifacts injection.

As explained previously at the beginning of this section, we wish to measure the sensitivity to the classifier models w.r.t. an eventual artificial distortion on the original document image with a series of artifacts, which are noise, contrast, brightness, focus blur, motion blur, brightness, or a combination of some of the previously cited. Here, 1000 images were selected, and different types of distortion artifacts (see Table 4) were added to them. Each level of artifacts, e.g., the amount of blur, is described in Table 4; this individual level is expressed in Figure 15 by the concept “class number” on the plots’ x-axis in Figure 15. This means that by increasing the “class number,” the amount of that specific artifact will correspondingly increase. For example, blur class 1 corresponds to kernel size 1 × 1, and blur class 10 corresponds to the kernel size of 19 × 19. Then, our classifier model is used to classify the distorted test images with the mentioned method. Figure 15 is showing the effects of different artifacts’ injection levels on the classifier’s performance. One does see that blur has a higher impact on decreasing the classifier’s accuracy. Therefore, considering this knowledge about the negative impact of blur on classification performance, deblurring document images before classifying them can significantly increase classification accuracy. In Figure 16, our novel model is compared to a selection of the best of related works. We do see in Figure 16 that our model is clearly more robust in the presence of the different levels of artifacts compared to the other models involved in the benchmarking of Figure 16. 

## 5. Conclusions

In this paper, a new deep neural model for document image classification was developed and studied comprehensively. The main challenges faced in this paper can be summarized into two different parts: (a) detecting a document within a given input document image and then extracting the document image; and (b) classifying the document under hard conditions related to artifacts contamination, a high number of classes, and strong similarities between some of the classes. Different models were developed progressively and tested until we reached and created our best model for our usage under the hard conditions at the table. For increasing the overall performance of the novel final model under the real hard conditions, the novel model was trained in the form of adversarial training, whereby artificial artifacts such as a blur, noise, and contrast changes were being injected into the training data. The validation and the consecutive benchmarking results show very promising results by outperforming the latest models for different performance metrics such as accuracy and precision. Despite the much better classification performance, the processing time remains in the same range as for the other competing classifier models. 

The positive effect of the preprocessing layer was also justified by involving the so-called NMI scores metrics (see Table 1). As we can see for model III, the NMI score improved respectively is much higher. Therefore, using the preprocessor layer helps our classifier solve problems by highlighting the edges (through the Sobel filter) and other interesting parts (through the Gabor filter). 

On the other hand, involving the Resnet 101 with a “feature fusion” provides a robust classifier integrated with the first classifier. The fusion of the two classifiers (see Model V) is essentially an excellent classifier combination for robustly and reliably classifying any document types.

For practical use of our novel developed model in visual sensors, one can increase the accuracy of the model by adding adaptability and self-adjustment through different methods such as “Adaptive Recognition” [61], “Dynamic Identification” [62], and “Manipulator controls” [63]. 

Regarding significantly reducing the negative sensitivity of our classifier to distortions in the input document image, a realistic strategy can include dirty training; this means to have a significant amount of virtually and naturally distorted training datasets besides the clean document images. A data augmentation concept involving GAN (generative adversarial networks) will be (in future works) developed to generate a dirty dataset of document images that reflect the real distortions of document images captured from mobile phones. 

## Figures and Tables

**Table 1:** The effect of adding specific features on NMI Scores. The values obtained for the input features for the various deep-learning models used (for the test datasets used in this work).

*Model*	CNN Model without Multi-Layer Channels—Model I (Figure 5)	CNN Model with Some Multi-Channel Features—Model II (Figure 6)	CNN Model with More Multi-Channel Features—Model III (Figure 8)
*NMI*	83.3%	86.38%	91.22%

**Table 2:** Comparison of our novel model’s classification performance through different traditional metrics.

*Model*	Model I (Figure 5)	Model II (Figure 6)	Model III (Figure 8)	Model IV (Figure 10)	Model V (Figure 11)
*Accuracy*	84.5%	87.8%	90.8%	93.3%	96.3%
*Precision*	85.0%	87.6%	91.2%	94.1%	96.4%
*F1 Score*	84.4%	87.4%	90.8%	93.9%	96.3%
*Recall*	84.6%	87.1%	90.9%	93.7%	96.3%

**Table 3:** Classification accuracy of our CNN model compared to other classifier models known from the relevant literature. A total of 160,000 samples were used, whereby 40,000 samples were used for validation and 40,000 samples were used for testing the model.

Model	Accuracy	FNR	Processing Time (for a Single Input Image)
Small holistic CN [59]	85.00%	15.51%	9 ms
Holistic CN [59]	89.80%	11.82%	9 ms
Ensemble of CNN [59]	89.03%	12.20%	9 ms
AlexNe [60]	90.04%	10.51%	8 ms
GoogleNe [60]	88.40%	12.80%	10 ms
VGG-1 [60]	91.01%	8.56%	11 ms
Resnet-5 [60]	91.13%	9.75%	12 ms
C-SV [13]	92.20%	7.60%	3950 ms
DAE CNN [7]	90.20%	9.81%	20 ms
RCN [15]	88.50%	11.30%	18 ms
Our Model III	90.80%	10.21%	11 ms
Our Model IV	93.32%	6.92%	14 ms
**Our Model V**	**96.36%**	**4.33%**	**15 ms**

**Table 4:** Different artifacts related parameter ranges used for stress-testing (as shown in Figure 14) our classifier’s sensitivity.

**Artifact**	Generator	Description
Noise	$\begin{array}{l} Image_{output}=Image_{Input}\;Norm\left(0,\sigma \right)\\ where\;\;\sigma \in \{-0.5,-0.4,-0.3,-0.2,-0.1,\;0,\;0.1,\;0.2,\;0.3,\;0.4,\;0.5\} \end{array}$	Norm is a gaussian random generator with mean value zero and a variance of σ
Contrast	$\begin{array}{l} Image_{output}=Image_{Input}\;\;R\\ where\;R\;\in \left\{0.5,0.6,0.7,0.8,1.0,1.1,1.2,1.3,1.4,1.5\right\} \end{array}$	R is a real value from the given set
Brightness	$\begin{array}{l} Image_{output}=Image_{Input}+R\\ where\;R\;\in \left\{-128,-102,-76,-51,-25,\;0,\;25,\;51,\;76,\;102,\;128\right\} \end{array}$	R is a real value from the given set of values
Focus Blur	$\begin{array}{l} Image_{output}=Image_{Input}*Kernel_{Focus\;Blur}\\ where\;KernelSize\;\in \left\{1,\;3,\;5,\;7,\;9\;,\;11\;,\;13,\;15,\;17,\;19\;\right\} \end{array}$	The Kernel matrix is defined for convolution with the original image.
Motion Blur	$\begin{array}{l} Image_{output}=Image_{Input}*Kernel_{Motion\;Blur}\\ \;where\;KernelSize\;\in \left\{1,\;3,\;5,\;7,\;9\;,\;11\;,\;13,\;15,\;17,\;19\;\right\} \end{array}$	The motion blur kernel has one direction of 45 degrees.

## Data Availability

Not applicable.
